# Severe Fever with Thrombocytopenia Syndrome in Patients Suspected of Having Scrub Typhus

**DOI:** 10.3201/eid2211.160597

**Published:** 2016-11

**Authors:** Yu Mi Wi, Hye In Woo, Dahee Park, Keun Hwa Lee, Cheol-In Kang, Doo Ryeon Chung, Kyong Ran Peck, Jae-Hoon Song

**Affiliations:** Sungkyunkwan University, Changwon-si, South Korea (Y.M. Wi, H.I. Woo);; Jeju National University School of Medicine, Jeju-si, South Korea (D. Park, K.H. Lee);; Sungkyunkwan University School of Medicine, Seoul, South Korea (C-.I. Kang, D.R Chung, K.R. Peck, J.-H. Song)

**Keywords:** SFTS and Suspected Scrub Typhus, severe fever with thrombocytopenia syndrome, scrub typhus, mite, tick, vector, vector-borne infections, severe fever with thrombocytopenia syndrome virus, viruses, bacteria, South Korea

## Abstract

To determine prevalence of severe fever with thrombocytopenia syndrome in South Korea, we examined serum samples from patients with fever and insect bite history in scrub typhus–endemic areas. During the 2013 scrub typhus season, prevalence of this syndrome among patients suspected of having scrub typhus was high (23.0%), suggesting possible co-infection.

Severe fever with thrombocytopenia syndrome (SFTS) is an emerging infectious disease caused by a novel phlebovirus in the family *Bunyaviridae* (*1*,*2*). The disease is characterized by fever, gastrointestinal signs and symptoms, leukopenia, and thrombocytopenia (*1*,*2*). Exposure to ticks, weeds, and shrubs have been found to be risk factors (*2*,*3*).

In South Korea, areas in which SFTS and scrub typhus are endemic overlap (*4*,*5*). Scrub typhus is caused by *Orientia tsutsugamushi*, which are bacteria transmitted to humans by chigger mite bites. Scrub typhus is a major public health problem during harvest season in South Korea (October and November); 10,485 cases were reported in 2013 (*5*). The clinical presentations of scrub typhus and SFTS are similar; signs and symptoms typically develop within 1–2 weeks of infection and include fever, headache, malaise, and gastrointestinal upset.

In South Korea, febrile patients with a history of bug bites are generally suspected of having scrub typhus and are prescribed antimicrobial drugs (e.g., doxycycline or azithromycin) in the early phases of the disease. We investigated the prevalence of SFTS in patients with fever and a history of insect bites in scrub typhus–endemic areas.

## The Study

During scrub typhus season (September–December) in 2013, we collected serum samples from 74 patients at Samsung Changwon Hospital (Sungkyunkwan University, Changwon-si, South Korea) who had fever and a history of bug bites. At the time of patient admission, we tested for antibodies against *O. tsutsugamushi* by using a commercial immunochromatography kit (SD Bioline Tsutsugamushi Assay; Standard Diagnostics, Yongin, South Korea). For molecular diagnosis of SFTS virus (SFTSV), we performed reverse transcription PCR of partial small RNA segments as previously described (*6*). We then performed sequencing by using a BigDye Terminator Cycle Sequencing Kit (PerkinElmer Applied Biosystems, Warrington, UK).

Characteristics of SFTSV-positive and -negative populations were compared by using the χ^2^, Fisher exact, 2-sample *t*, or Mann–Whitney U tests, as appropriate. Logistic regression was used to identify predictors of SFTS virus infection. Variables for which p value was <0.05 in univariate analysis were candidates for multivariate analysis. All analyses were conducted with SPSS for Windows version 18.0 (SPSS Inc., Chicago, IL, USA).

Among the 74 patients who had fever and a history of bug bites during scrub typhus season, the overall prevalence of SFTS infection was 23.0% (17/74). Detected SFTSV sequences showed 97.0%–99.0% identity with the partial sequence of the small RNA segment from SFTSV strains from South Korea (GenBank accession nos. KR612072–KR612088). No significant differences were found between the 2 patient groups (with and without SFTS) with regard to farming (Table). Patients infected with SFTSV were much older than those not infected. Among patients infected with SFTSV, clinical presentations of anorexia, nausea/vomiting, and a decreased level of consciousness were more prevalent; lactate dehydrogenase and C-reactive protein levels were remarkably higher; and albumin levels were lower than among patients without SFTSV infection. Incidence of lymphocytopenia was lower among patients with than without SFTSV.

**Table:** Demographic and laboratory characteristics of SFTS patients, South Korea, 2013*

Characteristics	SFTS PCR+, n = 17	SFTS PCR–, n = 57	p value
Male sex, no (%)	7 (41.2)	24 (42.1)	0.946
Age, mean ± SD	64.2 ± 15.5	54.5 ± 16.4	0.033
Farming, no (%)	12 (70.6)	29 (50.9)	0.151
Coexisting condition, no. (%)			
Chronic lung disease†	3 (17.6)	3 (5.3)	0.130
Chronic heart disease‡	6 (35.3)	11 (19.3)	0.197
Chronic renal disease	1 (5.9)	1 (1.8)	0.409
Diabetes	4 (23.5)	5 (8.8)	0.197
Chronic liver disease	1 (5.9)	5 (8.8)	0.580
Corticosteroid use	0	2 (3.5)	0.591
Cancer	0	3 (5.3)	0.451
Cerebrovascular disease	1 (5.9)	3 (5.3)	0.657
Clinical presentation			
Fever (temperature >38.3°C)	13 (76.5)	48 (84.2)	0.480
Headache	5 (29.4)	18 (31.6)	0.865
Myalgia	8 (47.1)	31 (54.4)	0.595
Anorexia	10 (58.8)	5 (8.8)	<0.001
Nausea/vomiting	8 (47.1)	10 (17.5)	0.022
Abdominal pain	2 (11.8)	5 (8.8)	0.657
Diarrhea	1 (5.9)	1 (1.8)	0.409
Cough	1 (5.9)	5 (8.8)	0.580
Dyspnea	1 (5.9)	2 (3.5)	0.549
Decreased consciousness	3 (17.6)	-	0.010
Rash	12 (70.6)	34 (59.6)	0.414
Laboratory findings at admission			
Leukopenia (<4,000 cells/mm^3^) no. (%)	2 (11.8)	18 (31.6)	0.131
Lymphocytopenia (<1,500 cells/mm^3^) no. (%)	6 (35.5)	43 (75.4)	0.002
Anemia (hematocrit <30%), no. (%)	3 (17.6)	4 (7.0)	0.341
Thrombocytopenia (<10^6^cells/mm^3^) no. (%)	7 (41.2)	13 (22.8)	0.221
CPK, IU/L, median (IQR)	67 (33–132)	76 (45–128)	0.512
LDH, IU/L, mean ± SD	533 ± 202	402 ± 151	0.021
AST, IU/L, median (IQR)	104 (48–194)	69 (54–112)	0.210
ALT, IU/L, median (IQR)	70 (30–119)	53 (35–83)	0.616
PT (INR), median (IQR)	1.08 (1.03–1.15)	1.03 (0.97–1.09)	0.057
CRP, mg/L, median (IQR)	71.6 (46.4–110.4)	42.9 (23.2–80.3)	0.034
BUN, mg/dL, median (IQR)	12.7 (9.3–18.2)	12.7 (9.4–15.3)	0.634
Creatinine, mg/dL, median (IQR)	0.9 (0.8–1.3)	0.8 (0.6–1.0)	0.510
Albumin, g/dL, mean ± SD	2.9 ± 0.7	3.3 ± 0.5	0.005
Hematuria, no. (%)	3 (20.0)	20 (35.7)	0.356
Outcome			
Intensive respiratory or vasopressor support	3 (17.6)	1 (1.8)	0.036
Time from symptom onset to admission, d, median (IQR)	3 (3–6.5)	4 (3–6)	0.599
Hospital stay, d, median (IQR)	0 (0–3)	1 (0–4)	0.432
Death, no (%)	1 (5.9)	0	0.230

*ALT, alanine aminotransferase; AST, aspartate aminotransferase; BUN, blood urea nitrogen; CPK, creatinine phosphokinase; CRP, C-reactive protein; INR, international normalized ratio; IQR, interquartile range; LDH, lactate dehydrogenase; PT, prothrombin time; SFTS, severe fever with thrombocytopenia syndrome.  †Asthma, chronic obstructive pulmonary disease, bronchiectasis. ‡Underlying coronary heart disease, chronic heart failure.

Multivariate analysis revealed that low albumin level at admission (odds ratio 0.19, 95% CI 0.04–0.99; p = 0.049) and anorexia (odds ratio 13.1, 95% CI 2.2–78.7; p = 0.005) were independent predictors of SFTS in patients suspected of having scrub typhus. The goodness of fit of the final logistic regression model seemed to be satisfactory (Hosmer-Lemeshow statistic, χ^2^ = 7.321; p = 0.396). Of the 17 SFTS patients, 4 had an *O. tsutsugamushi* antibody titer of 1:2,560, determined by immunochromatography at admission, and 3 showed seroconversion on paired serum samples. Those infected with SFTSV were more likely to require intensive respiratory or vasopressor support than those not infected. During hospitalization, 1 (5.9%) SFTS patient died. Length of hospital stay was similar between patients infected and not infected with SFTSV.

## Conclusions

In scrub typhus–endemic areas of South Korea, prevalence of SFTS among patients with suspected scrub typhus is quite high (23.0%). Independent predictors of SFTS in patients with suspected scrub typhus are low albumin level at admission and anorexia. Co-infection with SFTSV and *O. tsutsugamushi* was suspected for 7 patients. Patients with SFTS experienced a more severe clinical illness; however, outcomes such as death and length of hospital stay did not vary significantly between groups of patients with and without SFTSV.

As an emerging infectious disease, SFTS is an increasing public health threat because of its wide distribution and high mortality rate (*1*,*2*). In South Korea during April–December 2013, a total of 35 cases of SFTS were reported (*4*). The major signs and symptoms of these 35 patients were fever (100%), gastrointestinal upset (74%), fatigue (74%), thrombocytopenia (100%), and leukocytopenia (100%) (*4*). During 2013, the reported mortality rate among patients with SFTS in South Korea was 45.7% (16 deaths/35 patients), which is higher than that reported in China (6%–30%) (*1*,*2*). However, our study found a mortality rate of 5.9%, and illness was severe in only 3 (17.6%) patients. A large-scale serologic survey of 2,547 farmers living in rural areas of Jiangsu Province in China (*7*) found an overall SFTSV antibody prevalence rate of 1.30%, and the farmers seropositive for SFTS did not report having typical symptoms of SFTS. This finding suggests occurrence of asymptomatic or mild cases of SFTS. 

In our study, all SFTS patients initially received a diagnosis of scrub typhus because of its seasonality and the similar presentation of the 2 diseases. The Korea Center for Disease Control and Prevention and the National Notifiable Disease Surveillance System reported that during 2013, SFTS occurred mainly from April to September (*4*). However, we showed that SFTS was also prevalent in October and November (Figure).

**Figure:**
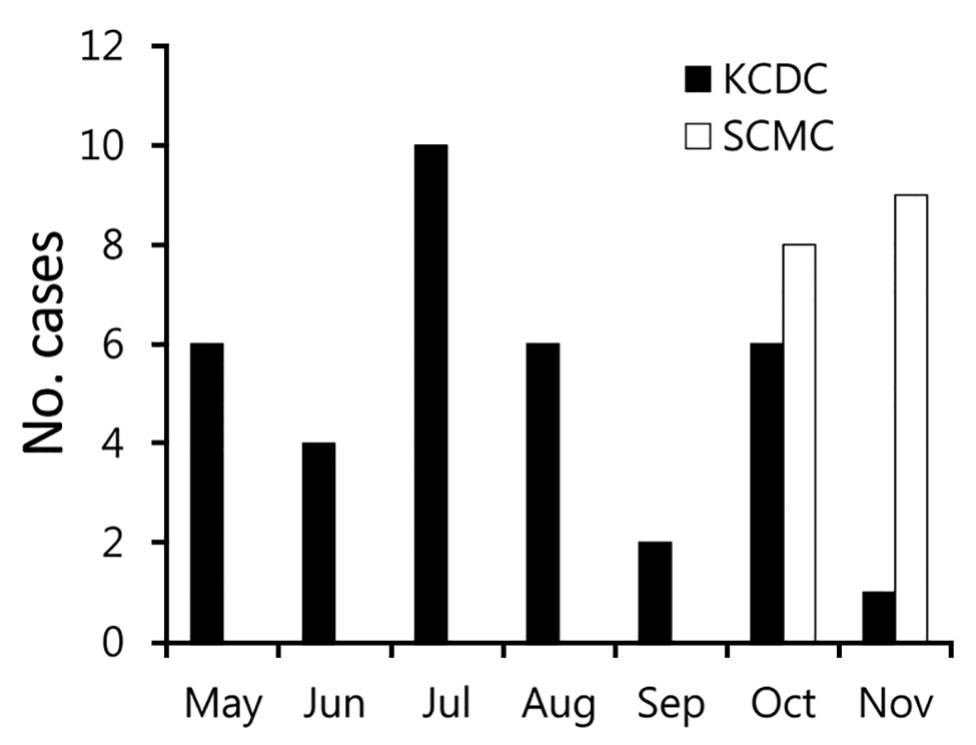
Seasonal distribution of severe fever with thrombocytopenia syndrome, South Korea, 2013. KCDC, Korea Center for Disease Control and Prevention; SCMC, Samsung Changwon Hospital.

SFTS is mainly transmitted to humans by SFTSV-infected ticks; however, person-to-person transmission by direct contact with infected blood or body fluids has also been reported (*8**,**9*). Therefore, standard precautions are necessary for healthcare workers in contact with patients with fever and a history of bug bites.

Previous studies have suggested that *Haemaphysalis longicornis* and *Rhipicephalus microplus* ticks are the most likely vectors of SFTSV transmission to humans (*10**,**11*). *H. longicornis* ticks are widespread in South Korea and their density is high during May–August, when temperatures are usually warm (*10*). In contrast, *Leptotrombidium scutellare* mites are major scrub typhus vectors with high density during autumn (scrub typhus season) (*12*). Therefore, for patients with fever and a history of bug bites, physicians in South Korea tend to suspect SFTS during summer and scrub typhus during autumn. In our study, the antibody titer of 1:2,560 in 7 patients suggests the possibility of SFTSV and *O. tsutsugamushi* co-infection. In China, SFTSV has also been detected by reverse transcription PCR in *L. scutellare* mites (*13*). Therefore, further research is needed to confirm the mite–SFTSV association in addition to the prevalence of SFTSV and *O. tsutsugamushi* co-infection.

The results of this study suggest that in South Korea, prevalence of SFTS is quite high among patients suspected of having scrub typhus. Signs and symptoms of SFTS can be atypical. Therefore, healthcare workers in contact with patients suspected of having scrub typhus should take standard precautions. Further epidemiologic research is needed to improve ability to accurately differentiate SFTS from other diseases and to confirm the vector of SFTS.
